# *UBE2L3* Polymorphism Amplifies NF-κB Activation and Promotes Plasma Cell Development, Linking Linear Ubiquitination to Multiple Autoimmune Diseases

**DOI:** 10.1016/j.ajhg.2014.12.024

**Published:** 2015-02-05

**Authors:** Myles J. Lewis, Simon Vyse, Adrian M. Shields, Sebastian Boeltz, Patrick A. Gordon, Timothy D. Spector, Paul J. Lehner, Henning Walczak, Timothy J. Vyse

**Affiliations:** 1 Department of Medical and Molecular Genetics, King’s College London, London SE1 9RT, UK; 2 Rheumatology Department, King’s College Hospital, London SE5 9RS, UK; 3 Department of Twin Research and Genetic Epidemiology, King’s College London, London SE1 9RT, UK; 4 Cambridge Institute for Medical Research, University of Cambridge, Cambridge CB2 0XY, UK; 5 Centre for Cell Death, Cancer and Inflammation, UCL Cancer Institute, University College London, London WC1E 6DD, UK

## Abstract

*UBE2L3* is associated with increased susceptibility to numerous autoimmune diseases, but the underlying mechanism is unexplained. By using data from a genome-wide association study of systemic lupus erythematosus (SLE), we observed a single risk haplotype spanning *UBE2L3*, consistently aligned across multiple autoimmune diseases, associated with increased *UBE2L3* expression in B cells and monocytes. rs140490 in the *UBE2L3* promoter region showed the strongest association. UBE2L3 is an E2 ubiquitin-conjugating enzyme, specially adapted to function with HECT and RING-in-between-RING (RBR) E3 ligases, including HOIL-1 and HOIP, components of the linear ubiquitin chain assembly complex (LUBAC). Our data demonstrate that UBE2L3 is the preferred E2 conjugating enzyme for LUBAC in vivo, and UBE2L3 is essential for LUBAC-mediated activation of NF-κB. By accurately quantifying NF-κB translocation in primary human cells from healthy individuals stratified by rs140490 genotype, we observed that the autoimmune disease risk *UBE2L3* genotype was correlated with basal NF-κB activation in unstimulated B cells and monocytes and regulated the sensitivity of NF-κB to CD40 stimulation in B cells and TNF stimulation in monocytes. The *UBE2L3* risk allele correlated with increased circulating plasmablast and plasma cell numbers in SLE individuals, consistent with substantially elevated UBE2L3 protein levels in plasmablasts and plasma cells. These results identify key immunological consequences of the *UBE2L3* autoimmune risk haplotype and highlight an important role for *UBE2L3* in plasmablast and plasma cell development.

## Introduction

*UBE2L3* is strongly associated with systemic lupus erythematosus (SLE) in genome-wide association studies and other genetic studies,^1–4^ as well as multiple autoimmune diseases (Table S1).^5–11^ UBE2L3 is an E2 ubiquitin-conjugating enzyme, also known as UbcH7. Although UBE2L3 was one of the first E2 conjugating enzymes to have its structure determined,^12^ its cellular functions have remained largely unknown. Because E2 enzymes appeared to be substitutable in ubiquitination assays, it was initially assumed that there was redundancy and lack of specificity between E2 enzymes. E2 enzymes have greater specificity than was first apparent, and they function with only selected E3 ligases in vivo. E2 enzymes have a critical role in determining ubiquitin (Ub) chain type.^13^ Because E2 enzymes act as ubiquitin shuttles, the kinetics of transfer of Ub from E2 to substrate in the case of RING E3 ligases, or onto the E3 in the case of HECT enzymes, might limit the speed of polyUb chain formation. Klevit and coworkers showed that UBE2L3 is incapable of conjugating ubiquitin onto free lysine and directly onto the target substrate, as is necessary for standard RING E3 ligases.^14^ UBE2L3 is therefore restricted to HECT-like E3s and co-operates with only a highly restricted set of dual RING E3 ligases with a RBR motif (RING-in-between-RING) and seven of the nine HECT E3 ligases.^15^

Linear ubiquitination, which involves sequential bonding of a ubiquitin moiety onto the Met-1 (M1) residue of ubiquitin,^16^ is mediated by the 600 kDa E3 complex LUBAC (linear ubiquitin chain assembly complex), composed of HOIL-1, HOIP, and Sharpin.^17–21^ LUBAC forms linear (M1) Ub chains on NEMO to activate the IKK complex. Deficiency of HOIL-1 or Sharpin inhibits phosphorylation and degradation of the NF-κB sequestration protein IκBα, leading to impaired activation of NF-κB. HOIL-1-deficient mice have defective NF-κB responses,^17^ and rare human loss-of-function mutations in HOIL-1 led to defective TNF signaling and abnormal IL-1 responses.^22^ Sharpin deficiency diminishes NF-κB activation, while increasing proinflammatory TNF-induced cell death, responsible for chronic proliferative dermatitis in Sharpin-deficient *Cpdm* mice.^19^ Thus LUBAC has been shown to be critical for NF-κB activation downstream of the TNF receptor 1 (TNFR1) and CD40. Presence of HOIP in B cells was necessary for CD40 signaling,^23^ and reduced immunoglobulin levels and impaired peritoneal B-1 cell development were observed in mice with conditional HOIP deficiency in B cells.^24^ HOIL-1 and HOIP are both RBR E3 ligases, so we hypothesized that UBE2L3 would be required for LUBAC to function in vivo. Recombinant HOIP and HOIL-1 or Sharpin can generate polyubiquitin chains with UBE2L3 in biochemical ubiquitination assays,^16,20^ although this might not be limited to UBE2L3, as hinted by the fact that the promiscuous E2 enzymes UBE2D1, UBE2D2, and UBE2D3 (UbcH5a, UbcH5b, and UbcH5c) can substitute for UBE2L3 in these assays.^16^ In this study we set out to investigate the relative importance of UBE2L3 to LUBAC function in vivo.

## Subjects and Methods

### Individuals and Genotyping

The study was approved by the UK National Research Ethics Service and institutional review boards of collaborators’ institutions prior to the commencement of the study. All study participants provided written consent at the time of sample collection. Samples from 4,946 individuals with SLE of European ancestry and 1,286 control subjects collected from multiple sites as part of an ongoing GWAS in SLE were genotyped on the Illumina Human Omni1-Quad BeadChip platform. Quality-control analysis of genotyping was carried out in accordance with Illumina’s Technical Note on Infinium Genotyping Data, excluding SNPs with poor clustering separation or call rate <0.95. Additional controls from the Health and Retirement study (dbGaP accession phs000428.v1.p1) genotyped on the Illumina Human Omni2.5-Quad platform were included in the analysis. Additional quality-control checks were made for individual missingness, SNP missingness, autosomal heterozygosity, identity-by-descent (via PLINK algorithm), Hardy-Weinberg equilibrium, and population structure (via EIGENSTRAT algorithm) with a panel of 50 ancestry informative markers. After quality-control analysis, 4,036 SLE-affected individuals and 6,959 control subjects with 696,085 SNPs were imputed with 1000 Genomes reference data via IMPUTE2.2 algorithm across region 21.60 Mb to 22.20 Mb on chromosome 22 spanning *UBE2L3*. To ensure reliability, imputed SNPs with an information score less than 0.9 were discarded. Single marker analysis was performed with SNPTEST (v.2.2) with four principal components as covariates in logistic regression. All SLE-affected individuals fulfilled the American College of Rheumatology (ACR) classification criteria for the diagnosis of SLE. SLE subphenotype data were available on a subset of 1,751 SLE-affected individuals. Case-case subphenotype statistical analysis was performed with SNPTEST with four principal components as covariates. Presence of lupus nephritis was defined by renal disorder subcomponent of the ACR diagnostic criteria.

### Expression Data

Microarray data from eQTL studies were obtained for lymphoblastoid cell lines from 270 HapMap individuals (GEO database GSE6536)^25^ and for CD19^+^ B cells and CD14^+^ monocytes isolated from peripheral blood mononuclear cells (PBMCs) from 288 healthy individuals (ArrayExpress E-MTAB-945).^26^ For both data sets, SNPs across region 21.60 Mb to 22.20 Mb on chromosome 22 were processed with IMPUTE2.2 to generate imputed genotype data at rs140490. For protein studies, healthy individuals, whose genotypes had been imputed at rs140490, were recalled from the TwinsUK resource. PBMCs were obtained from blood samples, and CD4^+^ T cells and CD19^+^ B cells were isolated by positive (Miltenyi) and negative (Invitrogen) selection, respectively, via magnetic beads and lysed with ice-cold RIPA buffer with protease and phosphatase inhibitors (Complete Mini and PhosSTOP, Roche). BCA assay (Pierce) was used to equalize total protein concentration. UBE2L3 protein levels were assessed by immunoblot. Densitometry was quantified with ImageJ software (NIH) normalized to actin.

### Plasmids and Cell Culture

The following plasmids on the pCMV6 vector were purchased from Origene: untagged UBE2L3, HOIP, UBE2D1, UBE2D2, UBE2D3, Sharpin-Myc-DDK, and empty vector (pCMV6-AC). pCMV6-HOIL-1-Myc-DDK (Origene) was subcloned into pCMV6AC to remove the C terminus Myc-DDK tag. pcDNA3.1-V5-His-HOIP and pcDNA3.1-V5-His-HOIP (p.Cys885Ser) were kind gifts of H. Walczak. pFlagCMV2-UbcH8 (UBE2L6) was obtained from Addgene. Variant p.Cys86Ser UBE2L3 was generated by site-directed mutagenesis (Stratagene Quikchange II XL). All plasmid ORF were verified by full sequencing. HEK293 cells (ATCC) were maintained in DMEM with 10% FBS.

### Antibodies

The following antibodies were used: anti-IκBα (C-21), rabbit anti-p65 (sc-372), anti-actin (sc-1616), and anti-UBE2L3 (C-20) from Santa Cruz; anti-Sharpin (4444), anti-p-IκBα (5A5), anti-JNK (56G8), anti-p-JNK (98F2), anti-ERK1/2 (137F5), anti-p-ERK1/2 (D13.14.4E), and anti-UBE2D3 (D60E2) from Cell Signaling; and anti-HOIL-1 (HPA024185, Sigma), anti-HOIP (SAB2102031, Sigma), anti-tubulin (AA13, Sigma), anti-UBE2L3 (20/UbcH7, BD Bioscience and A-640, Boston Biochem), anti-pan-UbcH5 (A-615, Boston Biochem), anti-FLAG (Sigma), and anti-V5-AlexaFluor647 (AbD Serotec). Mouse monoclonal anti-UBE2L3 antibody was conjugated to PE via Lynx rapid RPE conjugation kit (AbD Serotec) as per the manufacturer’s instructions.

### Luciferase Reporter Assay

GloResponse NF-κB-RE-*luc2P* HEK293 cell line (Promega) with stably integrated pGL4.32[luc2P/NF-κB-RE/Hygro] luciferase reporter were transiently transfected with combinations of plasmids via Fugene 6 (Promega). Luciferase activity was assayed by Luciferase Assay Reagent II (Promega) or One-Glo (Promega) on Berthold Orion luminometer, normalized to cell viability measured by CellTiterGlo assay (Promega).

### RNA Interference

GloResponse NF-κB-RE-*luc2P* HEK293 cells were seeded at 10,000 cells/well in 96-well plates. After 24 hr, cells were transfected with siRNA at a final concentration of 25 nmol/l with Dharmafect reagent #1. After 72 hr, cells were stimulated with TNF and analyzed by NF-κB luciferase reporter assay. For signaling pathway analysis, HEK293 cells at a density of 100,000 cells/well in 24-well plates were transfected as before and stimulated with TNF and lysed in the presence of protease and phosphatase inhibitor cocktail for immunoblot. Western blot gel electrophoresis, transfer, and detection was performed in parallel with identical film exposure duration. The following siRNA sequences were used: UBE2L3 sense 5′-CCGCAAAUGUGGGAUGAAA-3′, anti-sense 5′-UUUCAUCCCACAUUUGCGG-3′; HOIL-1 sense 5′-GCUCAGAUGCACACCGUCA-3′ and anti-sense 5′-UGACGGUGUGCAUCUGAGC-3′; HOIP sense 5′-GGCGUGGUGUCAAGUUUAA-3′ and anti-sense 5′-UUAAACUUGACACCACGCC-3′; and Sharpin sense 5′-CCUGGAAACUUGACGGAGA-3′ and anti-sense 5′-UCUCCGUCAAGUUUCCAGG-3′. siGENOME RISC-Free Control siRNA (Dharmacon) was used as a control.

### Real-Time Quantitative PCR

HEK293 cells were transfected with control, UBE2L3, or HOIP siRNA for 48 hr and stimulated with TNF 10 ng/ml for up to 6 hr. mRNA was isolated with Trizol reagent (Life Technologies), reverse transcribed into cDNA with SuperScript III First-strand Synthesis kit (Life Technologies), and quantified by real-time quantitative PCR with SYBR fast qPCR kit (Kapa) on an Applied Biosystems 7900HT instrument. Data normalized against β-actin levels were analyzed with Applied Biosystems RQ manager and GraphPad Prism software. The following gene specific primers were used: *NFKBIA* (IκBα) 5′-ATGCTCAGGAGCCCTGTAATG-3′ and 5′-CCCCACACTTCAACAGGAGT-3′; *CCL2* 5′-GAAAGTCTCTGCCGCCCTT-3′ and 5′-ATTGATTGCATCTGGCTGAGCG-3′; *TNFAIP3* (A20) 5′-GCGTTCAGGACACAGACTTG-3′ and 5′-TTCATCATTCCAGTTCCGAGTATC-3′; and *VCAM1* 5′-TTTGCAGCTTCTCAAGCTTTT-3′ and 5′-CCTGTGGTGCTGCAAGTC-3′.

### NF-κB Translocation

HEK293 cells were transiently transfected for 24 hr with HOIL-1, V5-His-HOIP, Sharpin, and/or UBE2L3. Cells were detached with TrypLE (Life Technologies), stimulated with TNF for 30 min, fixed with BD cytofix, permeabilized with 0.1% Triton X-100, and stained with rabbit anti-p65 (Santa Cruz), PE-conjugated anti-UBE2L3, and anti-V5-AlexaFluor647. Cells were washed, incubated with AlexaFluor488 F(ab)_2_ donkey anti-rabbit IgG (Jackson), washed, stained with DAPI. 20,000 cells per condition were acquired on Imagestream X imaging flow cytometer (Amnis). For ex vivo cell analysis, PBMCs were isolated via Histopaque from blood samples from previously genotyped healthy individuals (TwinsUK). CD19^+^ B cells were isolated by magnetic bead positive selection (Miltenyi) and CD14^+^ monocytes were isolated by negative selection (Miltenyi). Endotoxin-free MACS buffer was used throughout. Cell purity was confirmed by flow cytometry. B cells and monocytes were cultured in RPMI and stimulated with 0.1 μg/ml CD40L (Enzo) and 10 ng/ml TNF (Axxora), respectively, for up to 60 min. Cells were fixed and stained for p65 as above, washed, and stained with DRAQ5 (eBioscience), and 15,000–20,000 events per sample were acquired by Imagestream X. Data analysis was entirely automated with IDEAS software batch function applied to the entire cohort and performed fully blinded to genotype.

### Genotyping

Previously genotyped healthy twins were selected on the basis of imputed genotype at rs140490. Genotyping was confirmed in these individuals as well as in all SLE-affected individuals by TaqMan genotyping assay on Applied Biosystems 7900HT instrument.

### Flow Cytometry

Fresh PBMCs were isolated from blood obtained from 29 SLE-affected individuals and 25 healthy control subjects. Cells were stained with LIVE/DEAD Fixable Blue Dead cell stain (Invitrogen) to exclude dead cells, Fc receptor blocked (Human TruStain FcX, Biolegend), and surface-stained with the following markers: IgD-BrilliantViolet(BV)421 (IA6-2), CD19-BV510 (HIB19), CD27-BV650 (O323), CD138-FITC or CD138-PE-Cy7 (MI15), CD24-PerCP-Cy5.5 (ML5), CD95-PE-Cy7 (DX2), CD38-APC (HB7), CD20-APC-H7 (2H7) from Biolegend or BD. Cells were fixed with BD stabilizing fixative reagent. Cells were permeabilized with BD perm/wash buffer I and stained for UBE2L3-PE (described above) or permeabilized with Foxp3 permeabilization buffer (eBioscience) and stained for both UBE2L3-PE and Ki-67-Alexa488 (B56). Freshly stained cells were acquired on 5 laser BD SORP LSRFortessa instrument. BD CS&T beads were used immediately prior to every sample run to maintain instrument consistency throughout the entire study. Data were analyzed with FlowJo v.10 for the entire cohort by a single individual completely blind to genotype. Statistical analysis was performed with one-way ANOVA. SLE-affected individual demographics and disease characteristics are summarized in Table S2. On the same day that samples were drawn for flow cytometry, SLE disease activity was recorded with the SELENA modification of the SLEDAI. Measurement of erythrocyte sedimentation rate (ESR), anti-dsDNA antibody titer, and complement C3 and C4 levels were performed as part of routine clinical care.

## Results

### rs140490 Is Associated with SLE and the rs140409 Risk Allele Increases Expression of *UBE2L3*

**Figure 1:**
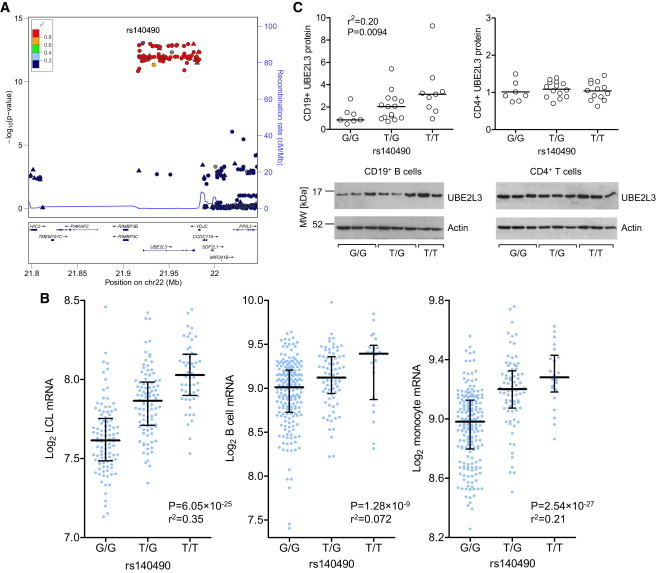
rs140490 Is Associated with SLE and a Cell-Specific Increase in UBE2L3 Production (A) Locuszoom plot showing SNPs around *UBE2L3* imputed to 1000 Genomes level. Recombination rate was calculated from HapMap data. (B) rs140490 is associated with increased expression in microarray data (expressed as log base 2, bars show median and interquartile range) from EBV-transformed lymphoblastoid cell lines, as well as increased mRNA expression in CD19^+^ B cells and CD14^+^ monocytes isolated from PBMCs. (C) Semiquantitative analysis of UBE2L3 protein level from immunoblot densitometry compared to actin, stratified by rs140490 genotype in CD19^+^ B cells and CD4^+^ T cells isolated from PBMCs from healthy individuals, with representative immunoblots shown from individuals for each rs140490 genotype.

*UBE2L3* genotype data from an ongoing SLE GWAS (T.J.V., data not shown) was imputed with 1000 Genomes reference data, identifying rs140490 as the most strongly associated SNP, located at −270 bp of the promoter region of *UBE2L3* (p = 8.6 × 10^−14^, OR 1.30, 95% CI: 1.21–1.39) (Figure 1A). Haplotype analysis from SLE-affected individuals shows that a single risk haplotype spans the entire gene (Figure S1A) and is the same risk haplotype across all autoimmune diseases (Figure S1B). Case-case subphenotype analysis available on a subset of 1,751 SLE case subjects demonstrated that rs140490 was associated with increased risk of lupus nephritis (p = 0.0036, OR 1.27, 95% CI: 1.08–1.49), suggesting that *UBE2L3* is associated with increased disease severity.

Genotypes from eQTL studies^25,26^ with microarray expression data for *UBE2L3* were imputed to 1000 Genomes level, showing that the rs140490 risk allele was strongly correlated with increased *UBE2L3* expression in EBV-transformed lymphoblastoid cell lines from HapMap individuals^25^ (p = 6.06 × 10^−25^). A similarly strong linear relationship was noted between alleles of rs140490 and *UBE2L3* expression in primary human B cells (p = 1.28 × 10^−9^) and monocytes (p = 2.54 × 10^−27^)^26^ (Figure 1B). Samples from genotyped healthy individuals (TwinsUK) confirmed that rs140490 increased UBE2L3 protein levels in CD19^+^ B cells isolated from peripheral blood (p = 0.0094) but that the risk SNP rs140490 did not significantly alter UBE2L3 protein levels in CD4^+^ T cells (Figure 1C). Consistent with this, eQTL microarray data on primary CD4^+^ T cells showed a lower p value (p = 4.4 × 10^−4^) compared to CD14^+^ monocytes (p = 3.4 × 10^−8^) for rs7444, which is in strong LD with rs140490, showing a reduced effect on *UBE2L3* expression in CD4^+^ T cells (data not shown).^27^

### UBE2L3 Exerts Rate-Limiting Control over LUBAC-Mediated NF-κB Activation

**Figure 2:**
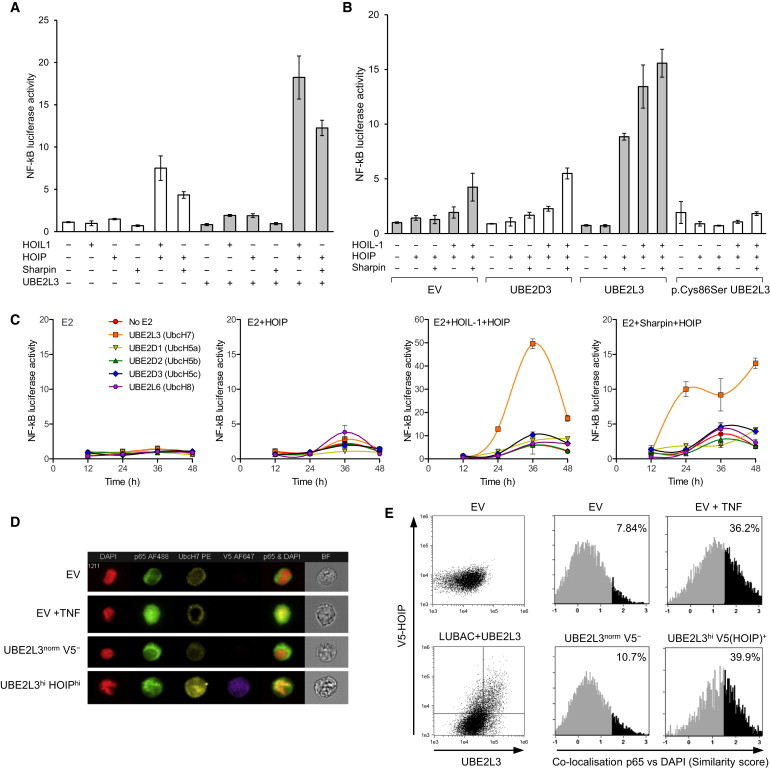
UBE2L3 Exerts Rate-Limiting Control over LUBAC-Mediated NF-κB Activation (A) Basal NF-κB luciferase activity in HEK293 cells in response to increased production of combinations of HOIL-1, HOIP, Sharpin, and UBE2L3. UBE2L3 enhances NF-κB activation due to increased levels of HOIL-1 and HOIP. Error bars represent standard error of the mean. (B) Dominant-negative variant p.Cys86Ser UBE2L3 suppresses NF-κB activity due to LUBAC. (C) UBE2L3 is the preferred E2 ubiquitin-conjugating enzyme for LUBAC in vivo. Luciferase assay time course over 48 hr in HEK293-NF-κB reporter cells transiently transfected different E2 ubiquitin-conjugating enzymes in combination with EV, HOIP alone, HOIL-1+HOIP, or Sharpin+HOIP, comparing the basal NF-κB response after increased production of individual E2 enzymes with LUBAC. (D and E) Comparison of NF-κB p65 translocation measured by Imagestream analysis in HEK293 cells transiently transfected with empty vector (EV) or HOIL-1+V5-HOIP+UBE2L3. p65 nuclear translocation is quantified by Imagestream similarity feature correlating fluorescence co-localization of AlexaFluor488-p65 with nuclear DAPI. Similarity histograms show levels of p65 translocation in EV, compared to unstimulated HEK293 cells transfected with HOIL-1+HOIP+UBE2L3. EV-transfected cells stimulated with TNF 10 ng/ml for 1 hr acted as a positive control. AlexaFluor647 anti-V5-tag and PE anti-UBE2L3 were used to gate cells with high levels of HOIP(V5) and UBE2L3, showing high p65 translocation in this group compared to cells with normal HOIP and UBE2L3 levels. Representative histograms are shown from one of three separate experiments. (D) Representative Imagestream images of HEK293 cells with median similarity level of p65 translocation as measured in (E) showing AlexaFluor488-p65 (green), DAPI nuclear dye (pseudocolored red), PE-UBE2L3 (yellow), and AlexaFluor647-V5(HOIP) (purple). Merged images show p65/DAPI overlap in yellow.

The effect of overproduction of UBE2L3 with different combinations of LUBAC components (HOIL-1, HOIP, Sharpin) was examined in NF-κB luciferase reporter cell line. Increased synthesis of UBE2L3 alone did not alter NF-κB activation, nor did increased production of UBE2L3 with HOIL-1, HOIP, or Sharpin as individual components of LUBAC (Figure 2A). In contrast, increased synthesis of UBE2L3 with any of the three known functional LUBAC combinations led to a substantial upregulation in NF-κB activity in unstimulated cells, compared to overproduction of LUBAC without UBE2L3 (Figures 2A and 2B). Dominant-negative variant p.Cys86Ser UBE2L3, which substitutes the catalytic cysteine required for ubiquitin binding, fully suppressed activation of NF-κB by LUBAC, which suggests that UBE2L3 is essential for LUBAC function (Figure 2B) and that LUBAC depends on UBE2L3 to activate NF-κB.

We compared UBE2L3 with other E2 enzymes to determine whether UBE2D1, UBE2D2, or UBE2D3 could regulate LUBAC-mediated activation of NF-κB, because these have been shown to polymerize ubiquitin with LUBAC in biochemical assays.^16,19^ UBE2L6 (UbcH8) was included because it is the only other human E2 other than UBE2L3 to possess a proline at position 87, suggesting adaptation to function with RBR E3 ligases.^14^ Increased production of HOIP with or without HOIL-1 or Sharpin was compared in the presence of different E2 conjugating enzymes (Figure 2C). In line with a specific role for UBE2L3 in enabling linear ubiquitination, increased synthesis of UBE2D1, UBE2D2, UBE2D3, or UBE2L6 did not affect NF-κB activation in the context of LUBAC, whereas substantial upregulation of NF-κB was seen with UBE2L3. This suggests that in vivo LUBAC has specific E2 requirements for NF-κB activation, with UBE2L3 exerting a much greater influence on LUBAC compared with the other E2 enzymes tested.

We sought to confirm that UBE2L3 in combination with LUBAC altered NF-κB p65 nuclear translocation. p65 translocation in HEK293 cells was quantified by imaging flow cytometry, using the similarity function to correlate co-localization of p65 with DAPI nuclear dye. In HEK293 cells transfected with HOIL-1, His-V5-HOIP, and UBE2L3, cells gated for high co-production of V5-tagged HOIP and UBE2L3 showed a substantial shift in the percentage of cells showing p65 nuclear translocation (39.9%), whereas no significant shift in p65 translocation (10.7%) was observed in cells with normal HOIP and UBE2L3 levels within the same sample (Figures 2D and 2E). This confirms that only those cells that produced high levels of both UBE2L3 and HOIP as the catalytic active component of LUBAC demonstrated a quantitative shift in p65 translocation in unstimulated cells.

### UBE2L3 in Combination with LUBAC Augments Late-Phase NF-κB Response to TNF

**Figure 3:**
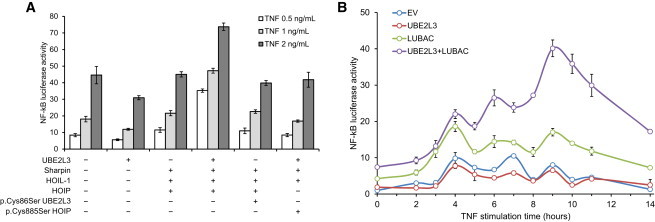
UBE2L3 and LUBAC Augment Late-Phase NF-κB Activation in Response to TNF (A) Increased production of LUBAC in combination with UBE2L3 augments NF-κB activation after 10 hr of TNF stimulation. This augmentation of the TNF response is abrogated by variant p.Cys86Ser UBE2L3 or variant p.Cys885Ser HOIP. (B) Time course showing NF-κB activation after TNF stimulation in response to increased synthesis of LUBAC and UBE2L3. Maximal dysregulation of NF-κB occurs in the late-phase response to TNF. Error bars represent standard error of the mean.

Although it is well documented that overproduction of LUBAC increases *basal* NF-κB activation, no studies have demonstrated that increased levels of LUBAC augment NF-κB activity *after* TNF stimulation. After stimulation by TNF at multiple doses, increased LUBAC and UBE2L3 synthesis resulted in much stronger NF-κB activation than LUBAC alone (Figure 3A). Furthermore, dominant-negative variant p.Cys86Ser UBE2L3 or p.Cys885Ser HOIP suppressed the increase in NF-κB activation seen in the context of LUBAC and UBE2L3. The amplification in NF-κB activation by LUBAC and UBE2L3 was noted to be greater at the late-phase component of the biphasic NF-κB activation response. Detailed time course analysis of TNF stimulation was performed, which showed that UBE2L3 and LUBAC maximally affected NF-κB activation at 9–10 hr (Figure 3B). It has been suggested that LUBAC is directly involved in activation of the IKK complex through linear ubiquitination of NEMO, leading to downregulation of IκBα and release of NF-κB during the initial activation phase of the NF-κB response.^17,19,20^ However, our data show that after TNF stimulation, overproduction of LUBAC and UBE2L3 has a more potent effect on late-phase NF-κB activation. Furthermore, dominant-negative variant p.Cys885Ser HOIP and dominant-negative p.Cys86Ser UBE2L3 did not completely suppress NF-κB activation, but reduced NF-κB activation to the level of response observed in control cells stimulated with TNF, in line with our previous observations on the effects of LUBAC deficiency.^18,19^

### Inhibition of UBE2L3 Impairs NF-κB Activation

**Figure 4:**
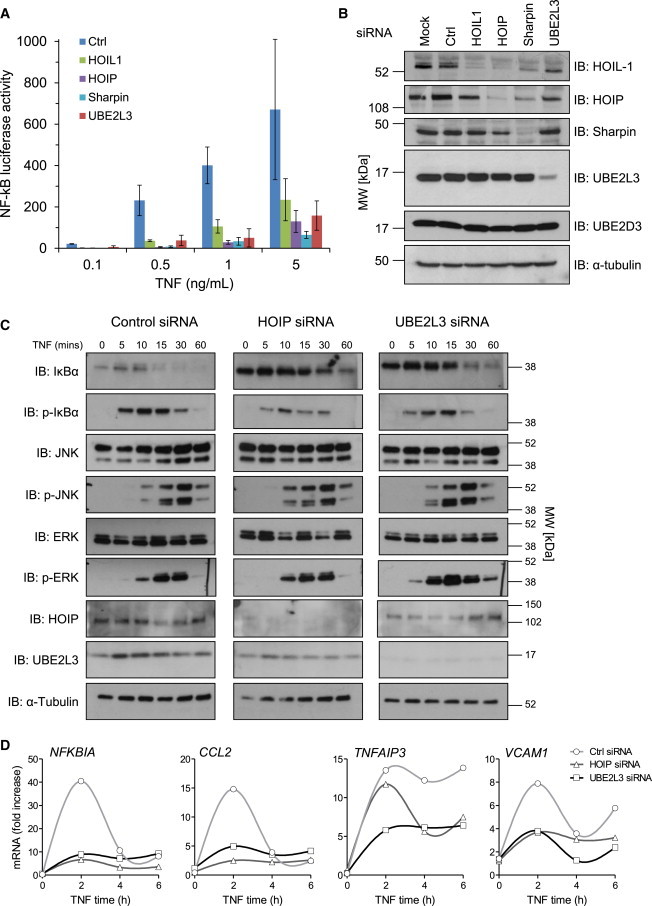
Inhibition of UBE2L3 Regulates NF-κB Signaling and Target Gene Transcription (A) Luciferase assay in HEK293-NF-κB reporter cell line showing that siRNA inhibition of HOIL-1, HOIP, Sharpin, and UBE2L3 inhibits NF-κB activation in response to TNF. Error bars represent standard error of the mean. (B) Immunoblot showing siRNA knockdown of HOIL-1, HOIP, Sharpin, and UBE2L3. (C) Inhibition of UBE2L3 or HOIP in HEK293 cells leads to basal increase in IκBα levels and impaired phosphorylation of IκBα in response to TNF, and subsequent IκBα degradation is reduced. UBE2L3 does not impair JNK or ERK phosphorylation. (D) Inhibition of UBE2L3 or HOIP reduces transcription of NF-κB target genes measured by real-time qPCR. HEK293 cells transfected with control, UBE2L3, or HOIP siRNA were stimulated with 10 ng/ml TNF.

To assess whether UBE2L3 is required for LUBAC-dependent NF-κB activation, we suppressed UBE2L3 via siRNA and observed that this indeed antagonized NF-κB activation (Figures 4A and 4B). Inhibition of UBE2L3 or HOIP led to basally increased IκBα levels in HEK293 cells. After TNF stimulation, inhibition of UBE2L3 or HOIP impaired phosphorylation and degradation of IκBα (Figure 4C). UBE2L3 inhibition exerted no effect on JNK or ERK phosphorylation. UBE2L3 blockade also inhibited TNF-induced gene transcription of *NFKBIA* (IκBα), *CCL2*, *TNFAIP3* (A20), and *VCAM1* measured by real-time PCR (Figure 4D), consistent with previous studies on LUBAC inhibition.^18,19^ Overall the results in Figures 2, 3, and 4 demonstrate that UBE2L3 is critical for LUBAC function and is the preferred E2 for LUBAC in vivo. The amount of UBE2L3 exerts rate-limiting control over LUBAC-mediated NF-κB activation, and together UBE2L3 and LUBAC play an important role in late-phase NF-κB activation in response to TNF.

### *UBE2L3* Genotype Influences Ex Vivo NF-κB Translocation

**Figure 5:**
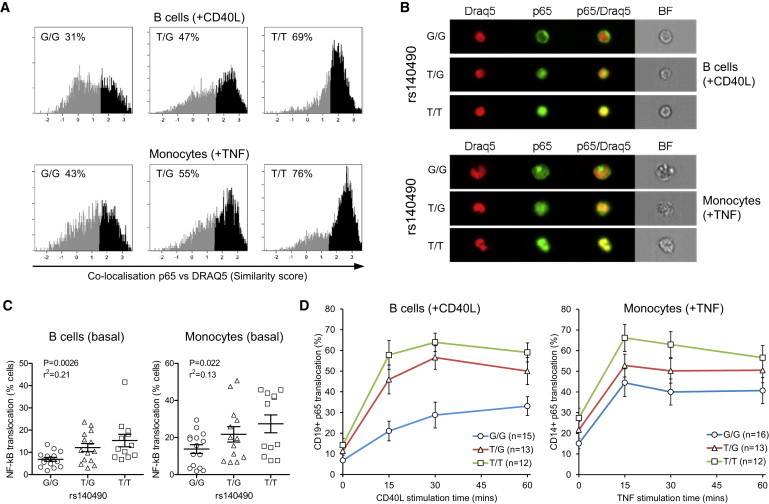
*UBE2L3* Genotype Affects Basal and Stimulated NF-κB Translocation in Primary Human B Cells and Monocytes Translocation of NF-κB p65 was quantified by similarity score on Imagestream analysis of CD19^+^ B cells and CD14^+^ monocytes isolated from PBMCs from healthy individuals, stratified by genotype at rs140490. (A) Representative similarity histograms measuring co-localization of p65 and nuclear dye DRAQ5 for B cells after 30 min of stimulation with CD40L and for monocytes after 30 min of TNF stimulation for each rs140490 genotype. Translocated cells were defined as similarity score > 1.5. (B) Imagestream images for each rs140490 genotype showing CD40L-stimulated B cells and TNF-stimulated monocytes of median similarity levels of p65 translocation from histograms in (A) showing p65 (Alexa488), DRAQ5 nuclear dye, and merged images. (C) Graphs show percent of NF-κB p65 nuclear translocated cells in unstimulated CD19^+^ B cells and CD14^+^ monocytes from genotyped individuals. (D) Graphs show 60 min time course of p65 translocation in response to stimulation of B cells with 0.1 μg/ml CD40L and monocytes with 10 ng/ml TNF, stratified by rs140490 genotype. Error bars represent standard error of the mean.

We postulated that it might be possible to detect the functional consequences on NF-κB from the genotypic effect of the *UBE2L3* risk haplotype at the cellular level in genotyped ex vivo cells. Using blood samples from healthy individuals stratified by *UBE2L3* genotype, we stimulated CD19^+^ B cells and CD14^+^ monocytes with CD40L or TNF, respectively, for up to 60 min. NF-κB p65 nuclear translocation was quantified by imaging flow cytometry, using the similarity feature (Figure 5A, representative images of B cells and monocytes in Figure 5B), demonstrating that nuclear translocation of p65 was strongest in the T/T genotype at rs140490 for both CD40L-stimulated B cells and TNF-stimulated monocytes. *UBE2L3*/rs140490 genotype was correlated with control over basal NF-κB activation in healthy human individuals for both B cells (p = 0.0026, r^2^ = 0.21) and monocytes (p = 0.022, r^2^ = 0.13) (Figure 5C), tending to a linear effect according to genotype, consistent with the effect of rs140490 on *UBE2L3* expression for these cell types. Similarly, time course analysis showed that rs140490 genotype affected the sensitivity of NF-κB to CD40 stimulation in B cells (genotype effect p = 0.00014; two-way ANOVA with repeated measures) and TNF stimulation in monocytes (genotype effect p = 0.0252) (Figure 5D).

### UBE2L3 Is Highly Abundant in Plasmablasts and Plasma Cells and *UBE2L3* Genotype Influences Plasmablast Proliferation in SLE

**Figure 6:**
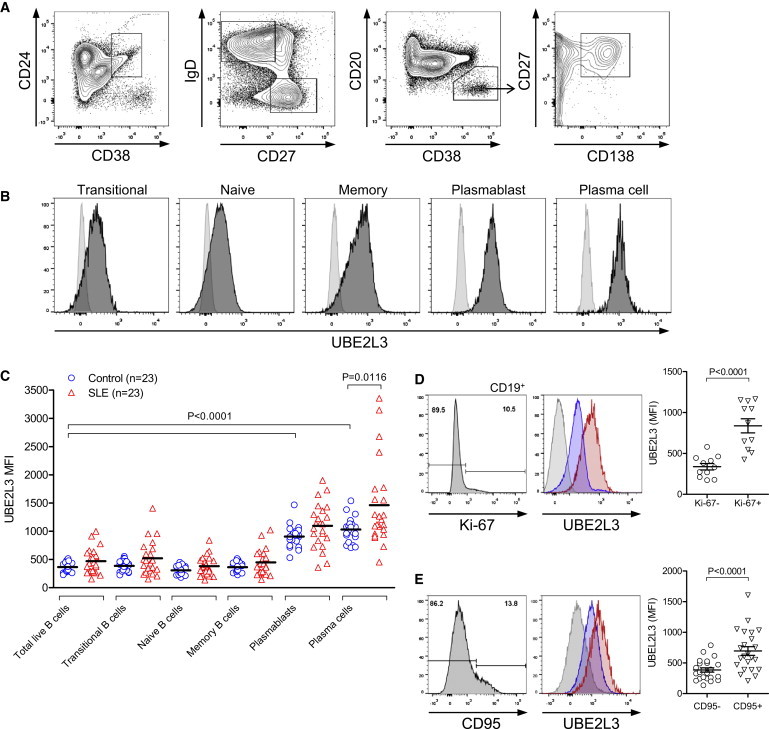
UBE2L3 Is Highly Abundant in Peripheral Blood Plasmablasts and Plasma Cells PBMCs isolated from healthy individuals and SLE-affected individuals were subject to ten-color flow cytometry to analyze UBE2L3 levels in B cell subsets. (A) Gating strategy for B cell subsets. (B) Histograms of B cell subsets from a representative SLE-affected individual demonstrating clear shift in UBE2L3 protein levels in CD19^mid^CD20^−^CD27^hi^CD38^hi^IgD^−^ plasmablast and CD20^−^CD27^hi^CD38^hi^IgD^−^CD138^hi^ plasma cell populations (isotype control light gray). (C) UBE2L3 abundance was highly increased in plasmablasts and plasma cells compared to other B cell subsets in both SLE-affected individuals and controls. (D and E) UBE2L3 levels were significantly higher in (D) Ki-67^+^ proliferating B cells and (E) CD95^+^ activated B cells in SLE-affected individuals. Histograms: isotype control, light gray; blue, Ki-67^−^ or CD95^−^; red, Ki-67^+^ or CD95^+^. Error bars represent standard error of the mean.

Given the importance of NF-κB activation for multiple stages of B cell development and survival, we hypothesized that UBE2L3 protein levels might be differentially regulated across different B cell subsets. We measured UBE2L3 protein levels by intracellular flow cytometry in B cell subsets in blood samples from healthy individuals and SLE-affected individuals (Figures 6A–6C) and observed that UBE2L3 was 3- to 4-fold more abundant in circulating CD19^mid^CD20^−^CD27^hi^CD38^hi^IgD^−^ plasmablasts (p < 0.0001) and CD20^−^CD27^hi^CD38^hi^IgD^−^CD138^hi^ plasma cells (p < 0.0001) compared to transitional, naive, and memory B cells. UBE2L3 levels were significantly higher in plasma cells in SLE-affected individuals compared to controls (p = 0.012) (Figure 6C). UBE2L3 protein levels were strongly elevated in Ki-67^+^ proliferating B cells (p < 0.0001) (Figure 6D) and also increased in CD95^+^ (Fas/APO-1) activated B cells (p < 0.0001) (Figure 6E), consistent with a role for UBE2L3-regulated NF-κB activation in both B cell proliferation and activation.

**Figure 7:**
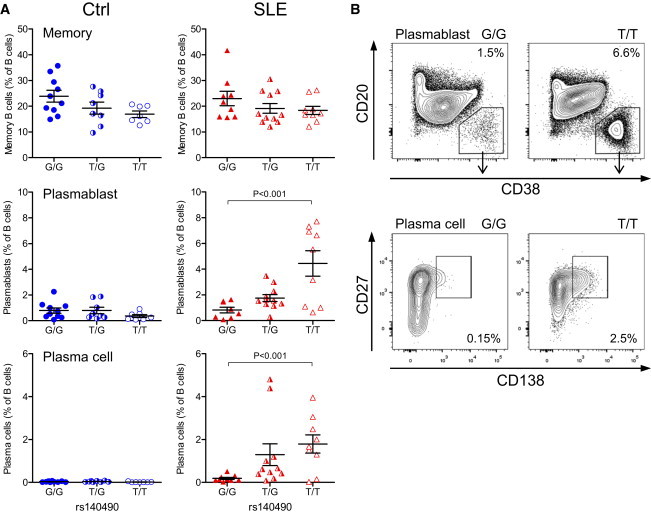
*UBE2L3* Genotype Influences Peripheral Blood Plasmablast Proliferation in SLE (A) The *UBE2L3* risk allele (rs140490 genotype) was associated with a significant expansion of peripheral blood plasmablasts and plasma cells (expressed as percent of live B cells) from SLE-affected individuals (n = 29), whereas this expansion was not seen in healthy controls (n = 25). Error bars represent standard error of the mean. (B) Representative flow cytometry plots showing percentages of plasmablasts and plasma cells (expressed as percent of live B cells) from SLE-affected individuals for each homozygous genotype.

Although rs140490 genotype did not affect naive, transitional, or memory cell population numbers in SLE-affected individuals, the *UBE2L3* risk allele was specifically associated with increased plasmablast and plasma cell number (both p < 0.001, one-way ANOVA) in SLE-affected individuals, but did not influence plasmablast or plasma cell numbers in healthy individuals (Figures 7A and 7B). *UBE2L3* genotype showed a non-significant trend to correlation with SLE Disease Activity Index (SLEDAI) recorded at the time blood was drawn for flow cytometric analysis (Figure S2A) (p = 0.063, one-way ANOVA). *UBE2L3* genotype was not correlated with blood markers of lupus activity including ESR, low serum complement C3 and C4 levels, or anti-dsDNA antibody or anti-nuclear antibody titer (Figures S2B–S2F). This suggests that the relationship between *UBE2L3* genotype and increased plasmablast and plasma cell numbers is not confounded by increased SLE disease activity. Taken as a whole, these results support the concept that by regulating basal and chronic low-level activation of NF-κB, the abundance of UBE2L3 might play a major regulatory role in abnormal B cell differentiation and proliferation in autoimmune diseases.

## Discussion

This study set out to understand the reasons behind the concomitant association between *UBE2L3* and multiple autoimmune diseases. Haplotype analysis shows that the *UBE2L3* locus has an unusually simple structure, with two haplotypes covering the majority of genetic variation and extending across the full length of the gene. Thus a single haplotype block is associated with SLE in our own data and the same risk haplotype is associated with multiple other autoimmune diseases (Figure S1). A recent GWAS of chronic hepatitis B in Han Chinese individuals found an association with *UBE2L3*.^28^ However, the *UBE2L3* haplotype associated with increased risk of chronic hepatitis B infection is consistently protective against autoimmune diseases (Figure S1), suggestive of a balanced polymorphism. The *UBE2L3* risk haplotype is associated with increased expression of *UBE2L3* in B cells and monocytes, which we have confirmed at the protein level in primary human B cells, but this appears to be cell specific, because rs140490 genotype had negligible effect on UBE2L3 protein levels in CD4^+^ T cells. Our data show that the SNP most strongly associated with SLE is rs140490 in the promoter region of *UBE2L3* 270 bp upstream of the 5′ start site. Given the location of rs140490, consistent with regulation of expression, it is biologically plausible that this might be the causal SNP. However, others have identified rs7444 in the 3′ UTR as the possible causal variant, and rs7444 is strongly correlated with rs140490.^4^

A major finding of this study is proof of the rate-limiting specificity of UBE2L3 in LUBAC-mediated NF-κB activation. UBE2L3 inhibition led to an upregulation in basal IκBα, impaired phosphorylation of IκBα, and delayed and reduced degradation of IκBα, similar to the pattern observed with HOIP inhibition. A recent study has independently shown that UBE2L3 inhibition reduced linear ubiquitination of NEMO and provides evidence confirming our observations that inhibition of UBE2L3 affects IκBα processing.^29^ In our study, the striking magnitude of the amplification of NF-κB activation in response to overproduction of UBE2L3 with LUBAC is in contrast to the data presented by Fu et al.,^29^ which showed that increased production of UBE2L3 alone exerted a modest effect on basal NF-κB activity. Our data contradict that of Fu et al. and show that increased UBE2L3 synthesis alone has no effect on NF-κB activity unless LUBAC components are also overproduced. This might be because HOIP in its native form exists in an autoinhibited state until complexed with HOIL-1 and Sharpin after TNF stimulation.^30,31^ We have also shown that dominant-negative p.Cys86Ser UBE2L3 fully suppressed NF-κB activation by LUBAC, confirming that UBE2L3 is critical for LUBAC function. By detailed analysis of the time course of NF-κB activation, we found that UBE2L3 in conjunction with LUBAC had a more potent effect in dysregulating the late phase of TNF-induced NF-κB activation, peaking at 9–10 hr, and that this effect could be suppressed by variant p.Cys885Ser HOIP, confirming that the effect of UBE2L3 on NF-κB is mediated through its interaction with HOIP. Although this is consistent with the current mechanism for LUBAC acting upstream of IκBα degradation, it raises the possibility that UBE2L3 and LUBAC affect late-phase negative-feedback inhibitors of NF-κB.^32^

Because UBE2L3 showed such a potent effect on NF-κB, we hypothesized that this could be detectable in response to *UBE2L3* genotype. We chose the p65 translocation assay as a measurable quantitative assay in close proximity to the direct molecular action of UBE2L3 and to reduce the possibility that genetic variation in proteins downstream of NF-κB would affect our ability to detect the functional consequences of rs140490. We observed that the risk allele at rs140490 was associated with increased p65 NF-κB translocation both at baseline in B cells and to a lesser extent in monocytes, but also in response to CD40L stimulation in B cells and TNF activation of monocytes. There are no known regulators of UBE2L3, but three deubiquitinating enzymes (DUBs) have been implicated in regulating linear ubiquitination, namely OTULIN,^33^ CYLD,^34^ and A20.^35,36^ A20 is encoded by *TNFAIP3*, which is associated with multiple autoimmune diseases,^37^ similar to *UBE2L3*. DUBs can directly regulate E2 enzymes,^38^ which raises the question as to whether OTULIN, CYLD, or A20 interact with UBE2L3.

We used PLINK EPISTASIS to investigate *UBE2L3* and *TNFAIP3* for SNP × SNP genetic interaction, analyzing SNPs that reached genome-wide significance for association with SLE. This analysis identified evidence of statistical interaction between rs140490 and two SNPs flanking *TNFAIP3*, namely rs80126770 (OR_interaction_ 1.28, p = 0.039) and rs6932056 (OR_interaction_ 1.26, p = 0.047). Both rs80126770 and rs6932056 tag the SLE-associated *TNFAIP3* risk haplotype with a functional TT>A polymorphic dinucleotide as described by Adrianto et al.^39^ Because of the strong LD across *UBE2L3*, other SNPs tagging the *UBE2L3* risk haplotype (including rs5754217 and rs7444) gave near-identical results for interaction with *TNFAIP3*.

This study shows the importance of *UBE2L3* genotype for both CD40-mediated B cell activation and TNF-mediated monocyte activation in primary human cells. Because aberrant autoreactive B cell survival is a key component of SLE pathogenesis, we postulated that *UBE2L3* genotype could influence B cell differentiation. Our data show that UBE2L3 protein levels are substantially elevated in plasmablasts, plasma cells, and Ki-67^+^ proliferating B cells (Figure 6). The *UBE2L3* risk haplotype correlated with increased plasmablast and plasma cell numbers in SLE-affected individuals (Figure 7), but not in healthy control subjects, which suggests that UBE2L3 plays an important role in plasmablast/plasma cell development in SLE. Dysregulated B cell development with increased numbers of circulating plasmablasts and CD138^+^ plasma cells is a well-recognized feature of SLE.^40^ Our data showing the effect of *UBE2L3* genotype on NF-κB activation in B cells and plasmablast and plasma cell numbers in SLE suggest that UBE2L3 has an important functional role in B cell proliferation and terminal B cell differentiation, consistent with the critical importance of NF-κB activation for B cell lymphoid development^41–43^ and plasma cell survival.^44^

In summary, our study shows that LUBAC-mediated activation of NF-κB is exquisitely sensitive to the expression level of *UBE2L3*, consistent with our finding that UBE2L3 is the preferred E2 for LUBAC in vivo. The molecular basis of the association of *UBE2L3* with numerous autoimmune diseases is mediated through the direct and measurable effect of the *UBE2L3* autoimmune risk haplotype on NF-κB both basally and in response to TNF and CD40L stimulation in primary human monocytes and B cells, respectively (Figure 5). In turn, *UBE2L3* risk alleles are strongly correlated with increased plasmablast and plasma cell numbers in SLE-affected individuals. The GWAS era has uncovered hundreds of disease-associated genetic variants, and yet very few studies have been able to identify functional consequences of complex trait genetic variants in ex vivo cells.

Because the tagging SNP rs140490 affects NF-κB responses in vivo and influences terminal B cell differentiation in SLE, we would predict that rs140490 could have potentially important clinical implications for prognosis in SLE, as well as response to biologic therapies such as anti-CD20 B cell depletion or anti-BLyS treatment. Because UBE2L3 is highly abundant in plasmablasts and plasma cells, our study provides primary evidence that UBE2L3 could potentially be a therapeutic target in SLE and possibly for other autoimmune diseases (Table S1) or plasma cell diseases such as multiple myeloma. A final important point can be inferred from the genetics of *UBE2L3*: the low-expressing *UBE2L3* genotype is not associated with ill health, which suggests that UBE2L3 inhibitors are likely to exhibit a safe window of tolerability.

## Supplemental Data

Document S1. Figures S1 and S2 and Tables S1 and S2

Document S2. Article plus Supplemental Data

## Web Resources

The URLs for data presented herein are as follows:

1000 Genomes, http://browser.1000genomes.org
dbGaP, http://www.ncbi.nlm.nih.gov/gap
Gene Expression Omnibus (GEO), http://www.ncbi.nlm.nih.gov/geo/
International HapMap Project, http://hapmap.ncbi.nlm.nih.gov/
TwinsUK, http://www.twinsuk.ac.uk/
