# Supplementary material for: UBE2L3 Polymorphism Amplifies NF-κB Activation and Promotes Plasma Cell Development, Linking Linear Ubiquitination to Multiple Autoimmune Diseases
Source: Am J Hum Genet. 2015 Feb 5;96(2):221–34. doi: 10.1016/j.ajhg.2014.12.024 (PMC4320258; doi:10.1016/j.ajhg.2014.12.024)
Supplement: Document S1. Figures S1 and S2 and Tables S1 and S2 [file mmc1.pdf]

The American Journal of Human Genetics
Supplemental Data

UBE2L3 Polymorphism Amplifies NF-κB Activation and Promotes Plasma Cell Development Linking Linear Ubiquitination to Multiple Autoimmune Diseases

Myles J. Lewis, Simon Vyse, Adrian M. Shields, Sebastian Boeltz, Patrick A. Gordon, Timothy D. Spector, Paul J. Lehner, Henning Walczak, and Timothy J. Vyse

Supplemental Data

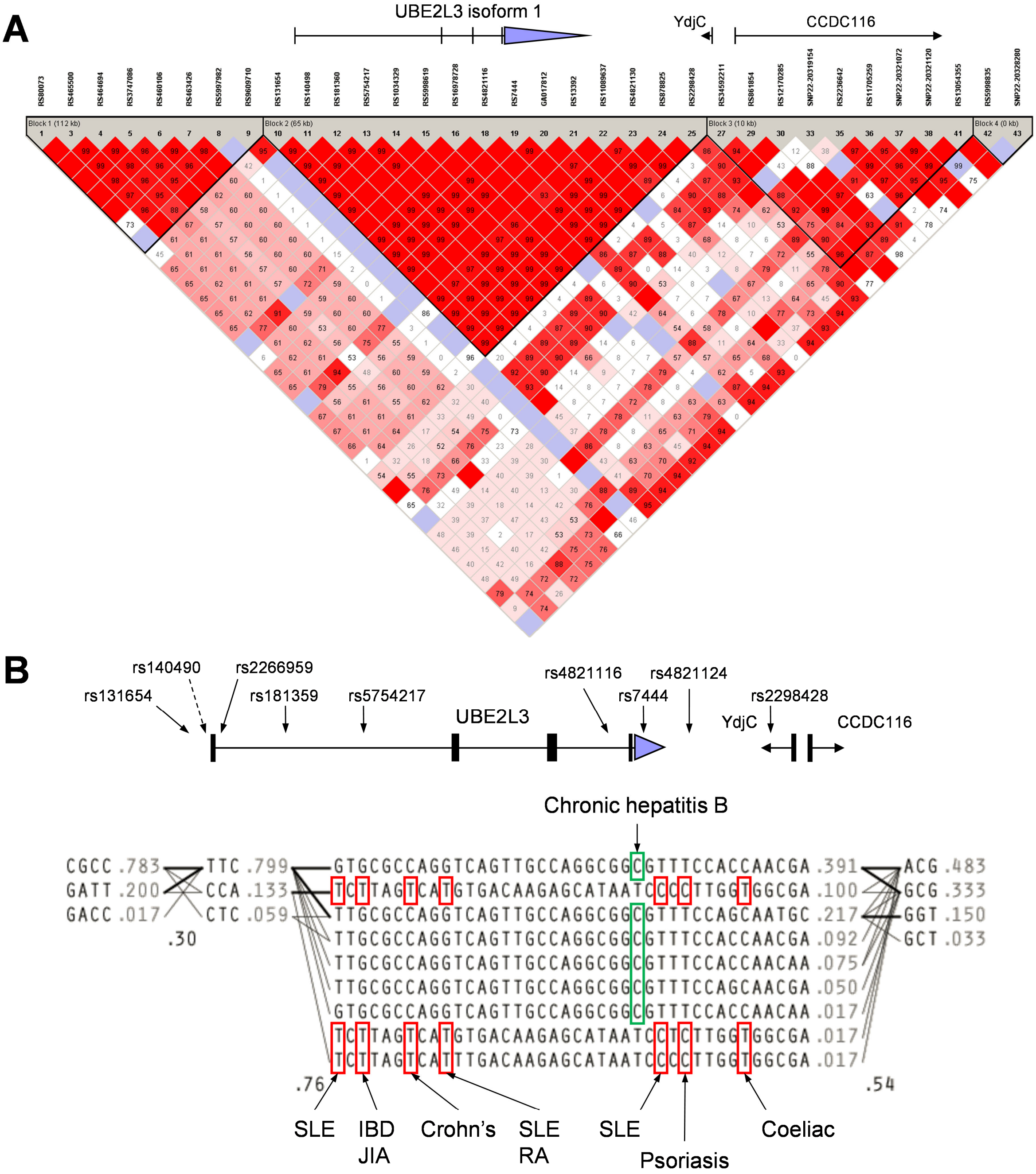

Figure S1. Single haplotype at *UBE2L3* is associated with increased risk of multiple autoimmune diseases.
(A) Linkage disequilibrium (LD) plot from haplotype analysis of SLE GWAS SNPs performed in Haploview showing LD encompasses the whole of *UBE2L3*. Squares colored to represent linkage disequilibrium measured as D'. (B) Haplotype analysis in Haploview showing SNPs from GWAS in multiple autoimmune diseases segregate on the same *UBE2L3* risk haplotype, whereas risk of chronic Hepatitis B carriage is associated with a haplotype which protects against autoimmune disease (SNP highlighted in green).

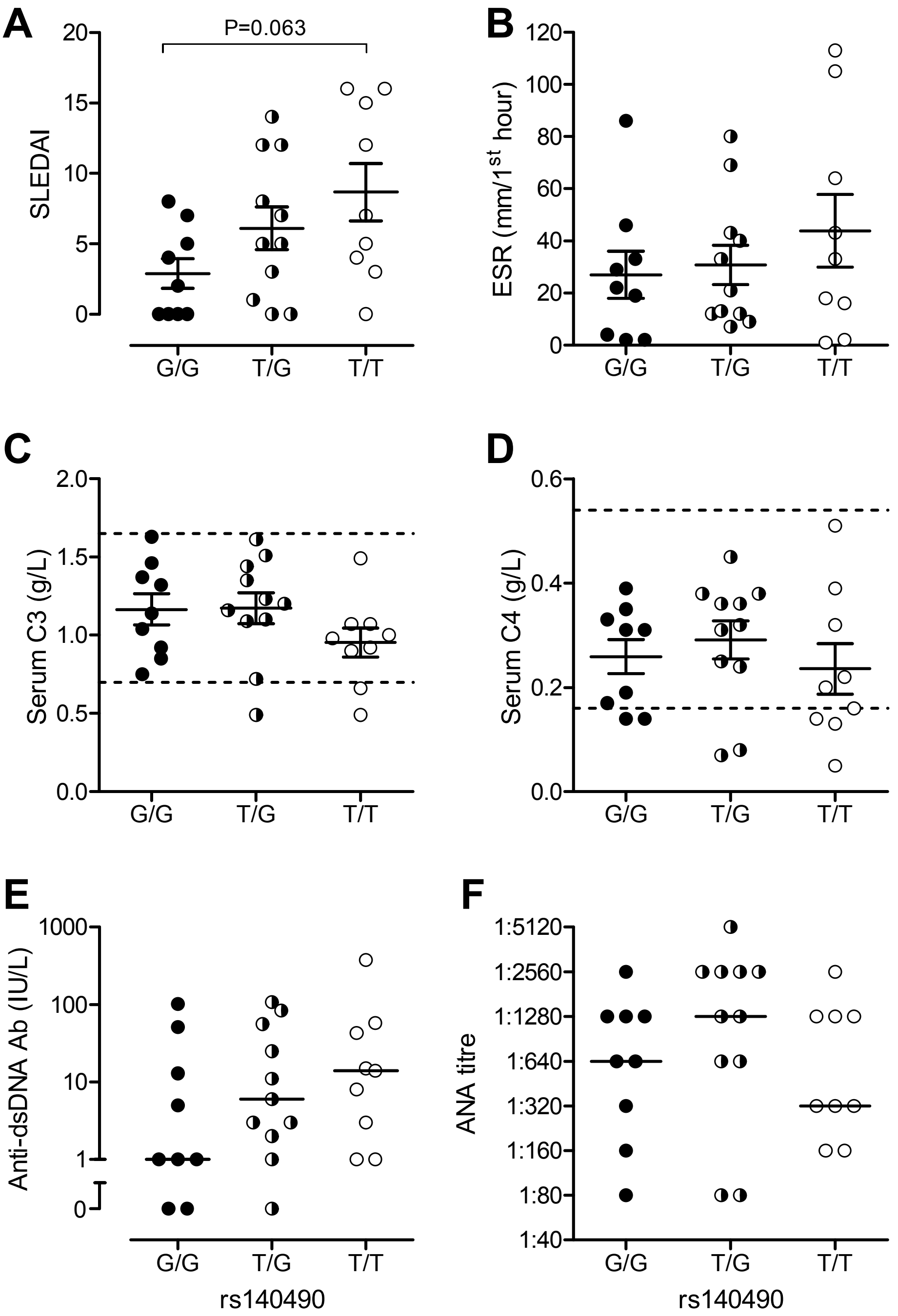

Figure S2. SLE disease activity data in SLE individuals compared with *UBE2L3* genotype.
(A) SLE Disease Activity Index (SLEDAI) was recorded for all SLE individuals on the same day that blood was drawn for flow cytometry analysis. (B-E) Blood markers of disease activity measured on the same day as blood samples were drawn for flow cytometry showing (B) Erythrocyte sedimentation rate (ESR), (C) serum complement C3 and (D) C4 levels and (E) anti-dsDNA antibody titer. Dotted lines in (C and D) represent laboratory normal range to show individuals with hypocomplementemia. (F) Maximum anti-nuclear antibody (ANA) titer recorded for each SLE individual as determined by case note review.

Table S1. Genetic studies showing an association between *UBE2L3* and autoimmune and infectious diseases.

SNP	Risk allele	Disease	Study design	OR (95% CI)	*P*	Reference
rs5754217	G→T	SLE	GWAS (SLEGEN)	1.22 (1.14-1.32)	7.53×10^{-8}	Harley et al, 2008
rs131654	C→A	SLE	GWAS	0.78 (0.74-0.83)	2.99×10^{-16}	Han et al, 2009
rs5754217	G→T	SLE	Replication study	1.20 (1.13-1.27)	2.3×10^{-6}	Gateva et al, 2009
rs7444	T→C	SLE	Multi-ethnic association study	n/a	2.21×10^{-14}	Wang et al, 2012
rs140490	G→C	SLE	GWAS	1.30 (1.21-1.39)	8.62×10^{-14}	Lewis et al*
rs5754217	G→T	RA	GWAS meta-analysis	1.10	4.8×10^{-5}	Stahl et al, 2010
rs11089637	T→C	RA	GWAS trans-ethnic meta-analysis	1.08 (1.05-1.11)	2.1×10^{-9}	Okada et al, 2014
rs2266959	T→G	JIA	GWAS	1.24 (1.15-1.33)	6.20×10^{-9}	Hinks et al, 2013
rs181359	G→A	Crohn's disease	GWAS meta-analysis	1.10 (1.06-1.15)	4.80×10^{-16}	Franke et al, 2010
rs2266959	T→G	IBD	GWAS meta-analysis	1.11 (1.07-1.15)	1.39×10^{-16}	Jostins et al, 2012
rs2298428	G→A	Celiac disease	GWAS	1.13 (1.08-1.19)	1.84×10^{-7}	Dubois et al, 2010
rs4821124	T→C	Psoriasis	GWAS (Immunochip)	1.13	3.8×10^{-8}	Tsoi et al, 2012
rs4821116	G→A	Hepatitis B	GWAS	0.82 (0.77-0.87)	1.71×10^{-12}	Hu et al, 2013

*The present study

Table S2. Disease characteristics of SLE individuals analysed by flow cytometry.

Individual number	Gender	Age	SLE disease characteristics	Serological status	Medications
1	F	41	Nephritis, hematologic	ANA 1:80, low C3, C4	HCQ
2	F	50	Arthritis, hematologic	ANA 1:1280, RNP, dsDNA, RF	HCQ
3	F	38	Photosensitivity, mouth ulcers, polyarthralgia, lymphopenia	ANA 1:80, Ro	Pred, MTX, HCQ
4	F	61	Arthritis, ILD, lymphopenia, photosensitivity	ANA 1:80, Ro	Pred, Aza
5	F	50	Arthritis, pleural effusion, ILD, lymphopenia	ANA 1:160	Pred, Aza
6	F	36	Malar rash, photosensitivity, arthritis	ANA 1:640, RNP, dsDNA	Nil
7	F	43	Nephritis	ANA 1:1280, dsDNA, low C3, C4	Ramipril
8	M	26	Malar rash, nephritis	ANA 1:1280, dsDNA, Ro, La, Sm, RNP, low C3, C4	Pred, MMF, HCQ
9	F	51	Discoid lupus, arthritis	ANA 1:5120, Ro, La, RNP, RF	HCQ
10	F	51	Discoid lupus, arthritis, ILD	ANA 1:160, Ro, Sm, RNP	Pred, MMF
11	F	39	Nephritis, alopecia	ANA 1:2560, Sm, RNP, dsDNA, low C3, C4	Pred, MMF, Everolimus
12	F	36	Nephritis, fetal loss	ANA 1:1280, Ro, La, RNP, low C3, C4	Pred, Aza, HCQ
13	F	61	Nephritis	ANA 1:160, Ro, RF	Aza, Pred, HCQ
14	M	45	Arthritis, nephritis	ANA 1:2560, RNP, dsDNA, low C3, C4	Depomedrone
15	M	33	Severe discoid lupus, alopecia, leukopenia	ANA 1:1280, dsDNA, Ro, Sm, RNP, low C3	Pred
16	F	46	TTP, ILD, AVN hip	ANA 1:1280, RNP	Quinine
17	F	33	Nephritis	ANA 1:320, Ro, La, dsDNA, low C3, C4	Aza, Pred, HCQ
18	F	52	Discoid lupus, alopecia, arthritis	ANA 1:2560, dsDNA	Mepacrine, HCQ, Aza,
19	F	44	Malar rash, pleural effusion, pericarditis, ILD	ANA 1:1280, Ro, RNP, dsDNA, low C3, C4	Pred, MMF
20	F	39	Digital vasculitis with ulceration, retinal vasculitis, PE	ANA 1:320, Ro, RNP, dsDNA, low C3, C4	Pred, tacrolimus, MPA
21	F	31	Discoid lupus, myositis	ANA 1:2560, Ro, Sm, RNP, dsDNA, low C3, C4	Pred, MMF, HCQ
22	F	25	SCLE, nephritis	ANA 1:640, Ro, Sm, RNP, dsDNA, low C3, C4	Pred, MTX, HCQ

Individual number	Gender	Age	SLE disease characteristics	Serological status	Medications
23	F	29	Polyarthritis, photosensitivity, malar rash, alopecia, Raynaud's	ANA 1:320, Ro, Sm, RNP, dsDNA, low C3, C4	Pred, HCQ
24	F	37	Nephritis	ANA 1:2560, Ro, RNP, dsDNA, low C3, C4	Pred, MTX, HCQ, rituximab
25	F	45	Autoimmune hepatitis, PHT	ANA 1:2560, dsDNA	HCQ, warfarin, bosentan
26	F	52	Inflammatory arthritis	ANA 1:640, Ro, RNP	Pred, MTX, HCQ
27	F	54	Thrombocytopenia, anaemia, neutropenia	ANA 1:1280, Sm, RNP	HCQ
28	F	63	Arthritis, diffuse rash	ANA 1:640, Ro, RNP	Pred, leflunomide, mepacrine
29	F	65	Polyarthritis, neutropenia	ANA 1:320, dsDNA	Pred, HCQ

Abbreviations: ILD – interstitial lung disease; TTP – thrombotic thrombocytopenic purpura; AVN – avascular necrosis; PE – pulmonary embolus; SCLE – subacute cutaneous lupus erythematosus; PHT – pulmonary hypertension; Aza – azathioprine; Pred – prednisolone; HCQ – hydroxychloroquine; MTX – methotrexate; MMF – mycophenolate mofetil; MPA – mycophenolic acid.
Serological status was determined by case note review and reports maximal anti-nuclear antibody (ANA) titer, previously documented anti-dsDNA or extractable nuclear antigen (ENA) autoantibody positivity and whether C3 and/or C4 hypocomplementemia were ever observed for each individual.
